# Prognostic Role of Chronic Obstructive Pulmonary Disease and Asthma Physiology Score for in-Hospital and 1-year Mortality in Patients with Acute Exacerbations of COPD

**DOI:** 10.1155/2022/4110562

**Published:** 2022-04-25

**Authors:** Zixiong Zeng, Qin Liu, Xiaoying Huang, Chunyan Lu, Juan Cheng, Yuqun Li, Guoping Hu, Liping Wei

**Affiliations:** ^1^Department of Respiratory Medicine, The Third Affiliated Hospital of Guangzhou Medical University, Guangzhou, Guangdong, China; ^2^State Key Laboratory of Respiratory Disease, National Clinical Research Center for Respiratory Disease, Guangzhou Institute of Respiratory Health, the First Affiliated Hospital of Guangzhou Medical University, Guangzhou, Guangdong 510182, China

## Abstract

Background and Objectives: Acute exacerbations of chronic obstructive pulmonary disease (AECOPD) often lead to high mortality. Chronic obstructive pulmonary disease and asthma physiology score (CAPS) is a simple clinical severity score. The aim of this study was to explore whether CAPS could be an effective predictor for in-hospital and 1-year mortality in AECOPD patients. *Methods*. We used CAPS to grade all patients and record their clinical characteristics. The receiver operator characteristic (ROC) curve was used to determine the cut-off of CAPS that discriminated survivors and non-survivors. Univariate and multivariate logistic regression analyses and Cox regression analyses were used to identify the risk factors for in-hospital and 1-year mortality, respectively. *Results*. 240 patients were enrolled in our study; 18 patients died during hospitalization and 29 patients died during the 1-year follow-up. Compared with in-hospital survivors, those who died were older (80.83 ± 6.06 vs. 76.94 ± 8.30 years old, *P* = 0.019) and had a higher percentage of congestive heart failure (61.1% vs. 14.4%, *P* < 0.001), higher CAPS levels (31.11 ± 10.05 vs. 16.49 ± 7.11 points, *P* < 0.001), and a lower BMI (19.48 ± 3.26 vs. 21.50 ± 3.86, *P* = 0.032). The area under the ROC curve of CAPS for in-hospital death was 0.91 (95% CI: 0.85–0.96) with a sensitivity of 0.889 and a specificity of 0.802 for a cut-off point of 21 points. CAPS ≥21 points was an independent risk factor for in-hospital mortality after adjustment for relative risk (RR) (RR = 13.28, 95% CI: 1.97–89.53, *P* = 0.008). Univariate Cox regression analysis showed that a CAPS ≥21 points (HR = 4.07, 95% CI: 1.97–8.44) was a risk factor for 1-year mortality. However, multivariate Cox regression analysis showed that CAPS (HR = 2.24, 95% CI: 0.90–5.53) was not associated with 1-year mortality. Conclusion: A CAPS ≥21 points was a strong and independent risk factor for in-hospital mortality in AECOPD patients and CAPS had no impact on the 1-year mortality in patients with acute exacerbations of COPD after discharge.

## 1. Introduction

AECOPD was defined as an acute worsening of the respiratory symptoms that results in additional therapy [1]. And AECOPD is an important event in the management of COPD because it negatively impacts the health status, rates of hospitalization and readmission, and disease progression. Improper management and untimely intervention of patients may cause an adverse hospitalization outcome and prognosis. To improve this phenomenon, it is essential for clinicians to assess the severity and prognosis of AECOPD patients in advance. Many studies have tried to explore the risk factors for mortality in AECOPD [2–4], and developed clinical prediction tools to improve prognostication in AECOPD [5–7]. However, many of these tools, such as APACHE II, APACHE III, and SAPS II, are difficult to use in clinical work because of their parameters. In addition, some severity scoring systems that could effectively reflect the severity of patients have not undergone external validation, which limits their widespread applicability [5, 8, 9] and there are still lacking of validated disease-specific scores to solve the problem. Wildman and colleagues [10] developed an acute physiology score named the chronic obstructive pulmonary disease and asthma physiology score (CAPS) for exacerbations of the obstructive airways disease. The score comprises eight indices (heart rate, mean arterial blood pressure, pH, sodium, urea, creatinine, albumin, and white blood count). From their study, the discrimination of CAPS for predicting hospital mortality exceeded that of the acute physiology scores from APACHE II and APACHE III, and they showed that CAPS could be used to estimate the prognostic impact of the physiological derangements accompanying an acute exacerbation of the obstructive airways disease. A study by Mucheng Zhang et al. [11] showed that CAPS was truly simple and useful in estimating the severity of patients with AECOPD complicated by type II respiratory failure. To date, few studies have focused on the association between CAPS and AECOPD. The utility of CAPS in hospitalized patients with AECOPD or whether it could be a robust prediction tool for the prognosis of AECOPD patients is still largely unknown. Therefore, a prospective study was designed to explore the predictive value of CAPS for in-hospital and 1-year mortality in AECOPD patients.

## 2. Methods

### 2.1. Study Design and Participants

All of the AECOPD patients admitted to the respiratory medicine department of the Third Affiliated Hospital of Guangzhou Medical University (Guangzhou, People's Republic of China) from September 20, 2016 to June 10, 2019 were screened; participants should be diagnosed with COPD previously according to the Global Initiative for Chronic Obstructive Lung Disease (GOLD) criteria [1]. Patients' primary diagnosis at admission must be AECOPD. Only the first admission could be included in the study. We recorded the worst values of the eight indices (heart rate, mean arterial blood pressure, pH, sodium, urea, creatinine, albumin, and white blood count) of all patients within 24 hours of admission to calculate the respective CAPS scoring systems [10] (Table 1), and patients were followed up every 3 months for 1 year by telephone after hospitalization.

The exclusion criteria were: interstitial lung disease, lung cancer, active pulmonary *tuberculosis* and other lung diseases, malignancy and not providing spirometry data. All patients provided written informed consent. The ethics committee of the Third Affiliated Hospital of Guangzhou Medical University approved the research proposal (No. 2016-004). The study was conducted in accordance with the Declaration of Helsinki.

### 2.2. Data Collection and Deﬁnitions

Both demographics of all subjects, including age, sex, the number of exacerbations in the previous year, comorbidities, and clinical data such as arterial blood gases (PaO_2_, PaCO_2_), routine blood test results (white blood cells, lymphocytes), and vital signs were collected. According to the reference value range of our hospital, lymphocyte count below 1.1 × 10^9^/L was defined as low lymphocyte count. On the basis of our previous article [12], the diagnoses of renal dysfunction and congestive heart failure would not be described again in this study.

### 2.3. Statistical Analysis

Normally distributed variables were presented as mean ± standard deviation (SD) and non-normally distributed variables were presented as medians (interquartile range, IQR). The comparisons of the two groups were using two-independent samples *t*-test and the Mann–Whitney *U* test, respectively. Categorical variables are summarized as numbers (%), and were analyzed using the chi-square test. A receiver operator curve was used for the threshold of CAPS. To analyze the risk factors of in-hospital mortality, logistic regression analysis was applied. Cox regression analyses were performed to evaluate the influence of CAPS levels on 1-year mortality and all variables detected in the univariate analyses with a *P* value of ≤0.2 were included in the multivariate analyses and some significant confounders (age, sex, smoking status, body mass index (BMI), GOLD stage, heart failure, and renal dysfunction) were also to be analyzed. Those lost to follow-up or with missing data would not be included in the analysis. According to different CAPS levels, Kaplan–Meier survival curves and the log-rank test were applied to compare the time to death. All analyses were two-sided, and *P* < 0.05 was considered to indicate statistical significance. Statistical analyses were performed with the use of SPSS 17.0 for windows (SPSS, Inc., Chicago, IL, USA).

## 3. Results

### 3.1. CAPS and In-Hospital Mortality

**Figure 1:**
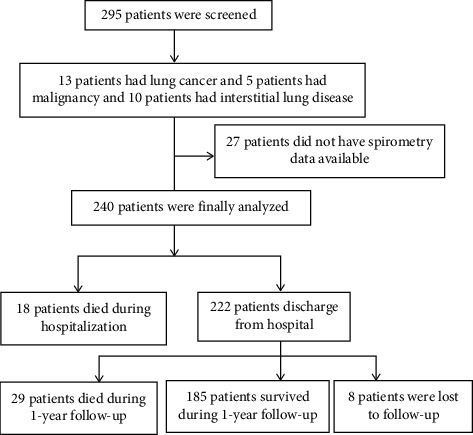
Flow chart of the study participants.

A total of 295 patients were recruited in this study and 240 patients with AECOPD were enrolled at last; (Figure 1) shows the flow chart of the participants. During the hospital stay, 18 subjects died and the differences between the survivor group and the non-survivor group during this period are shown in (Table 2). There were more patients who suffered from congestive heart failure and renal dysfunction in the non-survivor group than those in the survivor group. Those who died during hospitalization were older (80.83 ± 6.06 vs. 76.94 ± 8.30 years old, *P* = 0.019) than those in the survivor group and also had a lower BMI (19.48 ± 3.26 vs. 21.50 ± 3.86, *P* = 0.032), a lower lymphocyte count (0.93 ± 0.53 vs. 1.35 ± 0.76, *P* = 0.022), a higher percentage of neutrophils (83.8 ± 10.0 vs. 75.3 ± 11.8%, *P* = 0.003), a higher percentage of exacerbations during the preceding year (83.3% vs. 46.4%, *P* = 0.006), and had a higher CAPS level (31.11 ± 10.05 vs. 16.49 ± 7.11 points, *P* < 0.001) compared with the survivor group. Conversely, there were no signiﬁcant differences in sex, smoking status, smoking history, COPD stage, PH, PaO_2_, PaCO_2_, and the length of the hospital stay on comparing the non-survivor group with the survivor group.

### 3.2. ROC Curve Analysis to Predict In-Hospital Mortality

**Figure 2:**
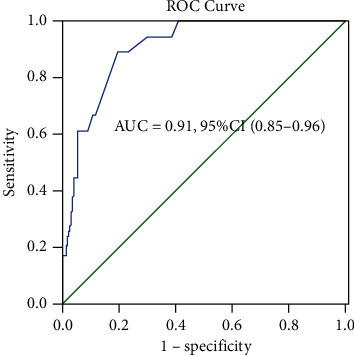
The ROC cure of CAPS as an overall predictor of death in patients.

(Figure 2) indicates that the area under the CAPS curve was 0.91 (95% CI: 0.85–0.96), and the cut-off value was 21 points with a sensitivity of 0.889 and a specificity of 0.802. Then, according to the CAPS levels, we divided the patients into two groups. There were 67 patients with CAPS levels ≥21 points and 173 patients with CAPS levels<21 points. As shown in (Table 3), there were signiﬁcant differences between the two groups regarding in-hospital mortality, age, congestive heart failure, renal dysfunction, number of acute exacerbations before admission, lymphocyte count, and percentage of neutrophils. However, there were no signiﬁcant differences between the two groups regarding sex, BMI, hospital stay, smoking status, smoking history, COPD stage, exacerbations during the preceding year, PH, PaO_2_, and PaCO_2_.

### 3.3. CAPS Levels and In-Hospital Mortality

The univariate logistic regression analysis showed that a decreased BMI, a history of exacerbations during the preceding year, a history of CHF, a history of RD, a CAPS ≥21 points, percentage of neutrophils ≥80%, a lymphocyte count ˂ 1.1 × 10^9^/L, pH < 7.35, pH > 7.45, and PaO2 < 60 mmHg were risk factors for in-hospital mortality and the best predictor of in-hospital mortality was a CAPS ≥21 points (RR = 26.82, 95% CI: 5.97–120.56, *P* < 0.001). A multinomial logistic regression analysis only indicated that a history of exacerbations during the preceding year, a history of CHF, and a CAPS ≥21 points were related to in-hospital mortality after controlling for age, sex, smoking status, and GOLD stage (Table 4). From the multinomial logistic regression analysis, a CAPS ≥21 points still remained a strong predictor of in-hospital mortality (RR = 13.28, 95% CI: 1.97–89.53, *P* = 0.008).

### 3.4. CAPS Levels and 1-Year Mortality

**Figure 3:**
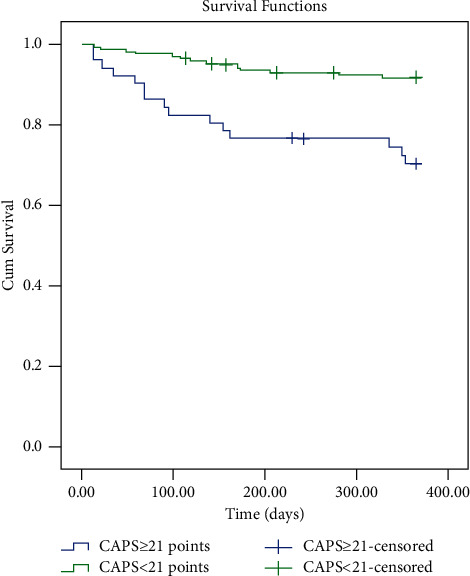
Kaplan–Meier survival curves evaluating the time to death in days for patients with CAPS ≥ 21 points and CAPS < 21 points.

A total of 29 patients died within 1 year and 8 patients were lost to follow-up. Based on the CAPS levels, Kaplan–Meier survival curves were applied to evaluate the time of death within 1 year (Figure 3). Compared to those patients with a CAPS <21 points, patients with a CAPS ≥21 points had an increased risk of 1-year mortality. Mean survival times for patients in the two groups were 295.84 days for the CAPS ≥ 21 points group and 346.05 days for the CAPS < 21 points group. At the meantime, the log-rank test showed that there was a signiﬁcant difference between the two survival curves (*P* < 0.001). The univariate Cox regression analysis showed that age, a history of CHF, and a CAPS ≥ 21 points (HR = 4.07, 95% CI: 1.97–8.44; *P* < 0.001) were risk factors for 1-year mortality (Table 5). Those variables with a *p*-value of ≤ 0.2 in univariate Cox regression analysis were also included in the multivariate Cox regression analysis and after controlling for some relevant covariates (sex, smoking status, BMI, COPD stage, and a history of RD), we found that age (per increase of 10-year), COPD stage, and a history of CHF were risk factors for 1-year mortality and CHF had an important impact on AECOPD patients' 1-year mortality(HR = 3.70, 95% CI: 1.52–9.02; *P* = 0.004). However, a CAPS ≥21 points was not associated with 1-year mortality (HR = 2.24,95% CI: 0.90–5.53, *P* = 0.082).

## 4. Discussion

From this study, we observed that patients who died in the hospital had higher CAPS levels than the survivors, and those who had a concentration of CAPS ≥ 21 points had an increased risk of in-hospital mortality. We also found that the CAPS ≥21 points was a risk factor for in-hospital mortality (multivariate analysis, RR = 13.28, 95% CI: 1.97–89.53, *P* = 0.008). Earlier we mentioned a study by Mucheng Zhang et al. reported that CAPS had good clinical value in assessing the condition of patients with AECOPD combined with type II respiratory failure and was better than that of APACHE II and APACHE III scores [11]. What's more, they also observed that in-hospital mortality had a positive correlation with CAPS. Our results were similar to theirs. However, their study was a retrospective study with only 82 AECOPD patients and did not explore the relationship between CAPS and the prognosis of AECOPD after discharge. Our study was a prospective study and we had reported the relationship between CAPS and the prognosis of COPD patients. In some ways, our study was a supplement to theirs. The variables of CAPS could truly reflect the physiological conditions of AECOPD patients during hospitalization, and it was relatively easy to operate compared with other clinical severity scoring systems [10]. In our research, we found that the area under the ROC curve of CAPS for in-hospital death was 0.91 (95% CI: 0.85–0.96, *P* < 0.001). Our results suggested that CAPS played a valuable role in predicting the in-hospital mortality of patients with AECOPD and might be a useful tool for clinicians to assess the severity of AECOPD patients and preliminarily determine whether the patients should be transferred to the ICU in our clinical work. Prognostic scores help identify those who are at a high risk of mortality in exacerbations of COPD. For example, DECAF score was developed for predicting the mortality of AECOPD and was superior to the other four scoring systems (CAPS, APACHE score, and BAP-65 score) [13, 14]. In addition, Echevarria et al. [15] obtained the same result. Although these instruments (excluding CAPS) showed good evaluation performance, their relatively higher number of variables make them difficult to operate and might limit their widespread applicability. All variables in CAPS were identified from the literature as having a potential to predict COPD survival [10]. Compared to those patients with a CAPS <21 points, patients with a CAPS ≥21 points had an increased risk of 1-year mortality and there was a signiﬁcant difference between the two survival curves (*P* < 0.001). However, the CAPS ≥21 points was not a risk factor of 1-year mortality multivariate analysis, (HR = 2.24, 95% CI: 0.90–5.53, *P* = 0.082). Two factors might explain this situation. One was that those who died during the follow-up period were significantly older (82.1 ± 6.8 vs. 76.2 ± 8.2 years old, *P* < 0.001) and had a history of heart failure (41.4% vs. 16.8%, *P* < 0.001) than those who survived. In previous studies [16–18], ageing and heart failure were significant and independent risk factors of all-cause mortality in COPD, influencing the course of COPD. To some extent, we considered that these factors might have weakened the effect of CAPS on 1-year mortality in multivariate COX regression analysis. Another factor was that a higher CAPS level meant that there were patients with a high risk of death, and most of them died during the hospitalization. They were rarely discharged to enter the follow-up process. The main purpose of this study was to explore the association between CAPS and in-hospital mortality. Moreover, the cut-off value of CAPS on admission that discriminated survivors and non-survivors was based on hospitalized patients rather than the patients followed-up in 1 year. These might be the main reasons for our results. However, this still needs to be explained by better predictive models in the future. In our study, patients with higher CAPS levels did have an increased risk of mortality during the follow-up period. Thus, we had better pay more attention to those patients with higher CAPS levels during hospitalization.

CHF was one of the most common comorbidities in COPD patients, and for AECOPD patients combined with heart failure, their prognosis is poor [19, 20]. A research by Boudestein LC et al. [21] found that the mortality of elderly patients with COPD combined with heart failure was higher than that of patients with COPD alone. In our study, stratified by CAPS, we observed that there was a signiﬁcant difference between the two groups regarding in-hospital mortality and we found that the CAPS ≥ 21 points group had more patients with congestive heart failure than the CAPS< 21 points group (32.8 vs. 12.1%, *P* < 0.001). The results suggested that the higher the CAPS levels, the higher the risk of heart failure in AECOPD patients. In our research, we found that CHF was an independent risk factor for in-hospital mortality (RR = 8.54, 95% CI: 1.67–43.67, *P* < 0.010). Furthermore, CHF was also an independent risk factor for 1-year mortality (HR = 3.70, 95% CI: 1.52–9.02; *P* = 0.004). The results were consistent with previous ﬁndings [12, 22]. Given that AECOPD patients with higher CAPS levels might have a higher risk of congestive heart failure, there might be a correlation between CAPS and congestive heart failure. Heart failure and hypoxia are common in COPD [23], leading to increased heart rate and blood pressure in COPD patients, causing higher CAPS levels.

A study from Hillas G et al. [24] reported that patients with COPD and especially with severe hypoxia and hypercapnia had a higher incidence of renal dysfunction and a higher risk of death than normal people. In our cohort, the incidence of renal dysfunction was higher in the non-survivor group than in the survivor group (8 (44.4%) vs. 19 (8.6%), *P* < 0.001). What's more, the CAPS ≥ 21 points group also had more AECOPD patients with renal dysfunction than the CAPS < 21 points group. However, in our research, both the multivariate logistic regression analysis and the multivariate Cox regression analysis showed that renal dysfunction was not a risk factor for in-hospital and 1-year mortality in AECOPD patients, which was inconsistent with Antonelli's results [8]. It should be noted that the incidence of COPD with renal dysfunction was low in our cohort and may cause the different results.

Hypercapnia was reported to be associated with a worse prognosis [25, 26]. Compared with in-hospital survivors, those who died seemed to be with a higher PaCO_2_ level (49.0 ± 22.3 vs. 44.9 ± 10.6). However, in our study, we have found that there was no signiﬁcant difference in PaCO_2_ levels between the two groups (*P* = 0.446). Additionally, we did not find hypercapnia to influence both in-hospital and 1 year mortality. We considered that our small sample size might be the main reason for the different results.

We also observed that the non-survivor group had a lower lymphocyte count than the survivor group. Furthermore, compared with the CAPS <21 points group, the CAPS ≥ 21 points group also had a lower lymphocyte count. The upper respiratory tract of patients with AECOPD was often infected by viruses [1] and most of the patients with hypoimmunity and poor basic health status might also have lower lymphocyte levels. Thus, we assumed that a lower lymphocyte count might reflect the conditions of AECOPD patients during hospitalization, and a study from China showed that a lower lymphocyte count was an independent risk factor for in-hospital mortality in patients with AECOPD [27]. Conversely, Our results were inconsistent with theirs. It should be noted that the threshold for the definition of low lymphocyte count was based on the reference range of our hospital and it might lead to different results, and more studies should be conducted on the association between lymphocyte count and in-hospital and 1-year mortality in patients with acute exacerbations of COPD.

Some limitations of our study should be mentioned. First, the exclusion of some patients due to insufficient clinical data could lead to selection bias and it was difficult to draw firm conclusions from the data for the relatively small number of subjects included in our study. Second, the intervention for COPD exacerbation was not identical for all AECOPD patients, which might be a confounder. Last, many outpatients already received oxygen therapy before the admission, which might have affected the data on blood gases. We hypothesized that these factors might affect the final results in our study. Thus, the relationship between CAPS levels and prognosis in COPD patients still needs to be further confirmed by large-sample, multicenter prospective studies.

## 5. Conclusion

In conclusion, we found that CAPS ≥21 points was a good predictor of disease severity in patients with AECOPD and the risk of in-hospital mortality. Perhaps clinicians could use a CAPS equal to or greater than 21 points as a reference limitation to initially assess the severity and the risk of death in hospitalized patients with AECOPD and intervene early to improve patient outcomes. However, further studies are still needed to verify the association between CAPS and long-term mortality.

## Figures and Tables

**Table 1:** The COPD and asthma physiology score.

Heart rate, min^−1^	<80	80–109	110–129	130–149	150–169	≥170	
Score	3	0	2	3	5	7	
MAP, mmHg	<40	40–49	50–59	60–69	70–89	90–99	≥100
Score	19	12	9	6	3	0	4
pH	<7.00	7.00–7.09	7.10–7.19	7.20–7.24	≥7.25		
Score	9	6	3	1	0		
Sodium, mmol l^−1^	<130	130–134	135–144	≥145			
Score	6	2	0	2			
Urea, mmol l^−1^	<2.5	2.5–6.7	6.8–11.9	12.0–17.9	≥18.0		
Score	0	8	16	22	24		
Creatinine, *μ*mol l^−1^	<150	150–199	≥200				
Score	0	5	8				
Albumin, g l^−1^	<15	15–19.9	20–24.9	25–29.9	30–34.9	≥35	
Score	20	14	8	6	4	0	
WBC, 10^9^ l^−1^	<4	4–14.9	15–19.9	20–24.9	≥25		
Score	7	0	1	4	7		

^*∗*^MAP, mean arterial pressure; WBC, white blood count.

**Table 2:** Baseline demographic characteristics of patients with AECOPD.

Characteristic	Dead (*n* = 18)	Alive (*n* = 222)	*P* Value
Age (years)	80.83 ± 6.06	76.94 ± 8.30	0.019
Sex, *n* (%)			0.868
Male	14 (77.8)	162 (73.0)	
Female	4 (22.2)	60 (27.0)	
BMI (kg/m^2^)	19.48 ± 3.26	21.50 ± 3.86	0.032
Hospital stay (days)	8.22 ± 4.78	7.66 ± 2.73	0.630
Smoking status, *n* (%)			0.273
Never smoker	2 (11.1)	57 (25.7)	
Current/ever smoker	16 (88.9)	165 (74.3)	
Smoking history (pack-years)	40.11 ± 27.59	36.67 ± 35.45	0.688
COPD stage, *n* (%)			0.939
I	1 (5.6)	19 (8.6)	
II	8 (44.4)	85 (38.3)	
III	6 (33.3)	82 (36.9)	
IV	3 (16.7)	36 (16.2)	
CHF, *n* (%)	11 (61.1)	32 (14.4)	<0.001
RD, *n* (%)	8 (44.4)	19 (8.6)	<0.001
Exacerbations during preceding year, *n* (%)	15 (83.3)	103 (46.4)	0.006
Lymphocyte count (×10^9^/L)	0.93 ± 0.53	1.35 ± 0.76	0.022
Percentage of neutrophils (%)	83.8 ± 10.0	75.3 ± 11.8	0.003
PH	7.36 ± 0.15	7.40 ± 0.04	0.280
PaO_2_ (mmHg)	85.0 ± 33.3	90.2 ± 25.7	0.425
PaCO_2_ (mmHg)	49.0 ± 22.3	44.9 ± 10.6	0.446
CAPS (points)	31.11 ± 10.05	16.49 ± 7.11	<0.001

^*∗*^CAPS, chronic obstructive pulmonary disease and asthma physiology score; CHF, congestive heart failure; RD, renal dysfunction; PaO_2_, arterial oxygen tension; PaCO_2_, arterial carbon dioxide tension.

**Table 3:** Baseline characteristics stratified by CAPS.

Characteristic	CAPS ≥21 points (67)	CAPS <21 points (173)	*P* Value
Hospitalization mortality (%)	23.88	1.16	<0.001
Age (years)	81.18 ± 6.79	75.71 ± 8.21	<0.001
Sex, *n* (%)			0.544
Male	51 (76.1)	125 (72.3)	
Female	16 (23.9)	48 (27.7)	
BMI (kg/m^2^)	20.92 ± 3.95	21.51 ± 3.81	0.288
Hospital stay (days)	8.09 ± 3.18	7.55 ± 2.81	0.204
Smoking status, *n* (%)			0.135
Never smoked	12 (17.9)	47 (27.2)	
Current smoker/ever smoked	55 (82.1)	126 (72.8)	
Smoking history (pack-years)	41.37 ± 37.68	35.21 ± 33.69	0.220
COPD stage, *n* (%)			0.228
I	3 (4.5)	17 (9.8)	
II	32 (47.8)	61 (35.3)	
III	21 (31.3)	67 (38.7)	
IV	11 (16.4)	28 (16.2)	
CHF, *n* (%)	22 (32.8)	21 (12.1)	<0.001
RD, *n* (%)	20 (29.9)	7 (4.0)	<0.001
Exacerbations during preceding year, *n* (%)	39 (58.2)	79 (45.7)	0.081
Number of acute exacerbations before admission (times)	1.0 (0, 2.0)	0 (0, 1.0)	0.047
Lymphocyte count (×10^9^/L)	1.07 ± 0.91	1.42 ± 0.65	0.001
Percentage of neutrophils (%)	82.3 ± 11.6	73.4 ± 11.1	<0.001
PH	7.39 ± 0.09	7.39 ± 0.04	0.642
PaO_2_ (mmHg)	88.3 ± 27.2	90.3 ± 26.0	0.592
PaCO_2_ (mmHg)	44.0 ± 13.2	45.7 ± 11.2	0.339

^*∗*^CAPS, chronic obstructive pulmonary disease and asthma physiology score; CHF, congestive heart failure; RD, renal dysfunction.

**Table 4:** Univariate and multivariate associations with in-hospital mortality.

Variable	Univariate analysis		Multivariate analysis	
	RR (95% CI)	*P* Value	RR (95% CI)	*P* Value
Age (per increase of 10-year)	1.93 (0.98–3.80)	0.057	0.43 (0.13–1.45)	0.173
Sex (female vs male)	1.30 (0.41–4.09)	0.658		
Smoking status (never smoker vs current/ever smoker)	2.76 (0.62–12.39)	0.184	1.32 (0.09–19.45)	0.838
BMI (per increase of 1 point)	0.86 (0.75–0.99)	0.035	0.87 (0.71–1.06)	0.155
COPD stage (per increase to next stage)	1.00 (0.57–1.76)	0.989		
Exacerbations during preceding year (yes vs no)	5.78 (1.63–20.52)	0.007	7.56 (1.33–42.91)	0.022
CHF (yes vs no)	9.33 (3.37–25.85)	<0.001	8.54 (1.67–43.67)	0.010
RD (yes vs no)	8.55 (3.02–24.23)	<0.001	4.15 (0.59–29.40)	0.154
CAPS (≥21 vs. <21 points)	26.82 (5.97–120.56)	<0.001	13.28 (1.97–89.53)	0.008
Percentage of neutrophils (<80 vs. ≥ 80%)	3.29 (1.19–9.12)	0.022	1.02 (0.16–6.45)	0.980
Lymphocyte count (<1.1 vs. ≥ 1.1 × 10^9^/L)	4.28 (1.47–12.46)	0.008	4.51 (0.85–23.99)	0.078
PH				
(PH < 7.35 vs.7.35 ≤ PH ≤ 7.45)	5.78 (1.73–19.37)	0.004	2.87 (0.37–22.12)	0.311
(PH > 7.45 vs.7.35 ≤ PH ≤ 7.45)	6.80 (2.00–23.10)	0.002	5.26 (0.93–29.77)	0.060
PaO_2_ (≥60 vs. < 60 mmHg)	4.97 (1.56–15.83)	0.007	1.40 (0.26–7.42)	0.694
PaCO_2_ (≥50 vs. <50 mmHg)	1.63 (0.58–4.58)	0.351		

^*∗*^95% CI, 95% Confidence Interval; RR, relative risk.

**Table 5:** Assessing the influence of CAPS levels on 1-year mortality by Cox regression analyses.

Variable	Univariate analysis		Multivariate analysis	
	HR (95% CI)	*P* Value	HR (95% CI)	*P* Value
Age (per increase of 10-year)	2.69 (1.54–4.68)	<0.001	3.08 (1.43–6.62)	0.004
Sex (female vs male)	1.45 (0.59–3.57)	0.415		
Smoking status never smoker vs current/ever smoker	1.67 (0.64–4.38)	0.296		
BMI (per increase of 1 point)	1.01 (0.92–1.11)	0.876		
COPD stage (per increase to next stage)	1.45 (0.94–2.23)	0.089	2.52 (1.28–4.95)	0.007
Exacerbations during preceding year (yes vs no)	1.23 (0.60–2.56)	0.572		
CHF (yes vs no)	4.84 (2.31–10.14)	<0.001	3.70 (1.52–9.02)	0.004
RD (yes vs no)	1.90 (0.66–5.45)	0.235		
CAPS (≥21 vs. < 21 points)	4.07 (1.97–8.44)	<0.001	2.24 (0.90–5.53)	0.082
Percentage of neutrophils (<80 vs. ≥ 80%)	1.98 (0.92–4.28)	0.083	0.82 (0.33–2.05)	0.672
Lymphocyte count (<1.1 vs.≥ 1.1×109/L)	1.23 (0.57–2.68)	0.598		
PH				
(PH < 7.35 vs. 7.35 ≤ PH ≤ 7.45)	0.77 (0.18–3.24)	0.718		
(PH > 7.45 vs. 7.35 ≤ PH ≤ 7.45)	1.34 (0.40–4.45)	0.634		
PaO2 (≥60 vs. <60 mmHg)	2.77 (0.96–8.05)	0.061	1.22 (0.38–3.93)	0.742
PaCO2 (≥50 vs. <50 mmHg)	1.50 (0.65–3.45)	0.341		

^*∗*^CHF, congestive heart failure; RD, renal dysfunction; HR, hazard ratio.

## Data Availability

All data relevant to the study are included in the article and are available from the corresponding author on reasonable request.
